# Immune-Mediated Inflammation Promotes Subclinical Atherosclerosis in Recent-Onset Psoriatic Arthritis Patients without Conventional Cardiovascular Risk Factors

**DOI:** 10.3389/fimmu.2018.00139

**Published:** 2018-02-26

**Authors:** Rodolfo A. Kolliker Frers, Vanesa Cosentino, Julia Tau, Eduardo M. Kerzberg, Adriana Urdapilleta, Monica Chiocconi, Nora Kogan, Matilde Otero-Losada, Francisco Capani

**Affiliations:** ^1^Laboratorio de Citoarquitectura y Plasticidad Neuronal (LCPN), Instituto de Investigaciones Cardiológicas, ININCA-UBA-CONICET, Buenos Aires, Argentina; ^2^Unidad de Reumatología, Hospital JM Ramos Mejía, Buenos Aires, Argentina; ^3^Laboratorio de Investigación Ocular, Departamento de Patología, Facultad de Medicina, Universidad de Buenos Aires, Buenos Aires, Argentina; ^4^Departamento de Radiología, Hospital JM Ramos Mejía, Buenos Aires, Argentina; ^5^Laboratorio de determinaciones hormonales, Sección de metabolismo de lípidos e hidratos de carbono, División Endocrinología, Departamento de Diagnóstico y Tratamiento, Hospital JM Ramos Mejía, Buenos Aires, Argentina; ^6^Sección Psoriasis, División Dermatología, Departamento de Clínica, Hospital JM Ramos Mejía, Buenos Aires, Argentina; ^7^Laboratorio de HPLC, Instituto de Investigaciones Cardiológicas, University of Buenos Aires, National Research Council, ININCA-UBA-CONICET, Buenos Aires, Argentina; ^8^Departamento de Biología, Universidad John F Kennedy, Buenos Aires, Argentina; ^9^Universidad Autónoma de Chile, Santiago, Chile

**Keywords:** atherogenesis, atherosclerosis, chronic inflammation, C-reactive protein, psoriatic arthritis, carotid intima–media thickness, IL-6, soluble intercellular adhesion molecule-1

## Abstract

Studies on the inflammatory burden in recent-onset psoriatic arthritis (PsA) patients without conventional cardiovascular risk factors (CVRFs) are not available. This preliminary study focuses on cardiovascular risk in cutaneous psoriasis (CPs) and recent-onset PsA patients. Blood biochemistry (glucose, cholesterol, uric acid, lipid profile and apolipoprotein B) was analyzed using standard kits. Proatherogenic inflammation markers, C-reactive protein (CRP) and interleukin-6 (IL-6), and endothelial activators monocyte chemoattractant protein-1 (MCP-1) and soluble intercellular adhesion molecule-1 (sICAM-1), were determined by enzyme-linked immunosorbent assay. Ultrasound images allowed measuring carotid intima–media thickness (cIMT). Our study first shows an increase in cIMT, and in serum levels of sICAM-1 and CRP in recent-onset PsA patients not presenting conventional CVRFs over the non-medicated time-period, from disease diagnosis to the beginning of pharmacological treatment, compared with healthy subjects. The outcome highlights the importance of monitoring serum level of sICAM1, CRP, and cIMT, and the value of primary prevention in psoriatic patients even with no history of cardiovascular events.

## Introduction

Psoriasis (Ps) and psoriatic arthritis (PsA) belong to the family of immune mediated inflammatory diseases (IMID) predominantly affecting skin and joints (1). Epidemiological studies show a peak incidence of Ps between the second and third decades of life (2) and a global prevalence around 2–3% regardless of sex (3). Approximately 7–42% of Ps patients develop inflammatory arthropathy as either mono or asymmetrical oligoarthritis (4).

The only blood biomarkers currently recommended in predicting cardiovascular risk are lipid-related indicators, however, lipid profile alone reflect only relative cardiovascular risk, as more than half of all cardiovascular events can occur in individuals with concentrations below the mean of total cholesterol (5).

The inflammatory background of atherosclerosis is undeniable (6). Epidemiological studies show that PsA is a high-risk factor for cardiovascular disease (CVD) (7–10) likely related to chronic inflammation leading to endothelial dysfunction (11) and plaque formation (12). Accelerated atherosclerosis has been linked to cutaneous psoriasis (CPs) according to its severity, even in the absence of joint symptoms (13).

Circulating markers of systemic inflammation could reliably anticipate cardiovascular morbidity and mortality (14). Soluble intercellular adhesion molecule-1 (sICAM-1), matrix metalloproteinase-3, N-terminal pro-B-type natriuretic peptide, interleukin-6 (IL-6), soluble CD40 ligand, and insulin-like growth factor binding protein-2, as well as age and diabetes mellitus, were found strongly associated with death and myocardial infarction (15).

On the other hand, similarities in immunopathogenic profile (Th1/Th17) between atherosclerosis (6, 16) and systemic inflammatory diseases like PsA and CPs have become an increasing topic of research. Therefore, systemic inflammatory markers such as C-reactive protein (CRP), interleukin-6 (IL-6) and soluble intercellular adhesion molecule-1 (sICAM-1) in combination with carotid intima–media thickness (cIMT) might be useful for cardiovascular risk (CVR) estimation (17, 18). Moreover, previous studies concluded that CRP, IL-6, and sICAM-1 showed more consistent results and independent predictive value than other inflammatory markers of atherogenesis (17). The contribution of IL-6 to the pivotal role of inflammation shared by psoriasis and atherosclerosis is crucial (19). Consistent with this point of view, CRP has been implicated in the pathogenesis of atherosclerosis (20) and appears to be not only a consistent predictor of cardiovascular events but an independent risk factor for myocardial infarction, stroke, and peripheral vascular disease as well (21). Of note, robust correlation reported for CRP serum levels with the Psoriasis Area and Severity Index (PASI) suggests that PASI is a measure of increased systemic inflammation rather than local skin inflammation (22). The isolated CRP level may be used in context with other CVR surrogate markers and clinical information to serve as a diagnostic tool (23).

The hepatic release of CRP increases the inflammatory burden in blood favoring endothelial wall injury (20). Whether the number and distribution of CRP receptors differ in PsA from those found in the general population requires more studies.

This study reports the noninvasive characterization of the immune-inflammatory profile associated with cardiovascular risk (CVR) in psoriatic patients not expressing the typical CVR factors. Inflammation was evaluated measuring endothelial and systemic markers, immunophenotype activation, inflammatory oxidation, and prothrombotic factors.

A rise in the blood level of sICAM-1 may reveal early proatherogenic alterations in the artery wall. The blood level of sICAM-1 has been related to the chronic and progressive inflammation underlying plaque formation in the coronary and carotid arteries appearing even decades before clinical CVD (24).

Among other markers, the monocyte chemoattractant protein-1 (MCP-1) has been implicated in atherosclerosis and coronary/peripheral arterial disease (24). It is a member of the C-C chemokine family produced by monocytes/macrophages, smooth muscle cells, endothelial cells (25–28), and adipocyte, and plays a key role in cardiovascular diseases. It is recognized by CCR-2 receptors on monocytes and serves as a chemotactic agent to recruit monocytes into the vascular wall (28). Pathogenesis and activity of psoriasis vulgaris may involve changes in MCP-1 blood levels (29). The level of systemic MCP-1 might provide information of local inflammation and has been proposed as a marker to assess disease severity in psoriatic patients (30).

To our knowledge, the relationship between cIMT, proinflammatory, and endothelial activation markers has not been thoroughly studied in recently diagnosed non-medicated PsA (29). This cross-sectional study compares the degree of atherogenesis based on subclinical parameters as cIMT, the presence of atherosclerosis plaque, and early inflammatory markers (CRP, IL-6, MCP1, and sICAM-1) in recent-onset PsA patients, low-intermediate CPs patients (control group accounting for inflammatory setting), and healthy subjects. Patients presenting conventional CVR factors or confounding comorbidities (diabetes) were excluded to avoid biased interpretation of data. Current evidence suggests that the inflammatory pathways involved in atherosclerosis modify peripheral blood levels of molecules derived from oxidative stress and vascular inflammation markers like CRP (31, 32), IL-6, membrane and soluble ICAM1 (ICAM1s) and MCP1 (30, 31). An important early step in the process of atherosclerosis is the adhesion of monocytes to activated endothelial cells, in which various adhesion molecules including ICAM1 are involved. TNFα, IL-1, IFNγ, and other cytokines induce endothelial ICAM1 expression (31, 33). Th1 secreting inflammatory cytokines also contribute to the pathogenesis of atherogenesis and psoriasis (34, 35).

## Subjects and Methods

### Study Design

Men and women between 25 and 75 years old attending the Hospital JM Ramos Mejía of Buenos Aires (HJMRM), Argentina, were recruited between April 2012 and December 2016. Following the selection process described below, 32 patients entered the study. Three groups were matched for age and body mass index (BMI): C (9 control healthy patients), CPs (9 low-intermediate CPs patients to account for psoriasis inflammation), and PsA (14 patients with recent PsA onset following low-intermediate CPs).

All participants signed written consent to join the study and completed a validated questionnaire. It conveyed anthropometric data, medical history, smoking status, dietary habits, alcohol consumption, exercise practice, and current medication for hypertension or diabetes.

The respective Institutional Ethics Committees of the Faculty of Medicine of the University of Buenos Aires (Comité de Bioética de la Facultad de Medicina de la Universidad de Buenos Aires) and of the Hospital JM Ramos Mejía (Comité de ética en investigación del Hospital José María Ramos Mejía del Gobierno de la Ciudad de Buenos Aires, Argentina, registered at The Office for Human Research Protections, NIH, USA Code FWA 00001767) approved the study protocol.

### Subjects’ Recruitment

Consecutive patients either satisfying the CASPAR classification criteria for psoriatic arthritis (36) or discarded from PsA diagnosis after rheumatologic evaluation were recruited at the Rheumatology Department and at the Dermatology Department and included in the PsA or the CPs group, respectively.

Before entering the study, both PsA and CPs cohorts were screened out for subclinical cardiovascular risk factors (CVRFs) or any ongoing inflammatory process. All participants underwent clinical and nutritional evaluation and a selection process based on the criteria shown below.

### Clinical Exclusion Criteria

Any of the followings were determinant for exclusion from the study:

- Concurrent disease.
  ◦ Chronic inflammatory or non-inflammatory illness, renal or hepatic failure.
  ◦ Metabolic syndrome (MetS) (37). Coexistence of at least 3 of the following: abdominal obesity, hyperglycemia, hyperinsulinemia or diabetes, hypertriglyceridemia or low HDL-C, and hypertension (38).
- Smoking history or current smoking (≥1 daily pack within 2 years before the study).
- Body mass index (BMI) <18.5 kg/m^2^ (underweight) or ≥30 kg/m^2^ (obesity).
- Alcohol consumption: more than 100 mL wine/day every day or equivalent.
- Treatment with anti-inflammatory drugs (NSAID), prednisone, TNF-blockers, disease-modifying antirheumatic drugs (DMARDs), hypocholesterolemic, or antidiabetic drugs.
- Cardiovascular history. Prior myocardial infarction, ictus, preexistent peripheral vascular disease, or so on.
- Hypertension. Diagnosed hypertension: SBP ≥ 130 mmHg/DBP ≥ 85 mmHg (38).
- Pregnancy.

### Biochemical Exclusion Criteria

Lipoproteins and other conventional CVR markers were measured, and associated CVR indices were calculated.

- Hyperglycemia either fasting > 140 mg/dL or postprandial > 200 mg/dL.
- Dislipemia (cholesterolemia > 240 mg/mL, HDL-C < 35 mg/100mL, LDL-C > 160 mg/dL, or TG ≥ 170 mg/dL).
- Triglyceridemia < 150 mg/dL
- Apolipoprotein B (ApoB) ≥ 130 mg/dL. Serum concentration of apolipoprotein was measured using nephelometry.

Of note, cutoff levels were set at ≥35 mg/dL for HDL cholesterol and >100 mg/dL for ApoB to avoid a substantial reduction in the number of patients in the study. Castelli index (total cholesterol/cholesterol HDLc) and the ApoB100/ApoA1 ratio were calculated to get the picture of the CVR relative to plasma atherogenicity. A Castelli index < 5 for men or <4.5 for women is related to a low CVR while higher values suggest a high CVR (39).

### Determinations

#### Psoriasis Activity and Severity

Disease activity score (DAS28) and Psoriasis Area and Severity Index (PASI) were calculated for PsA. For CPs, disease activity index was restricted to PASI (low: 0–20; mild: 21–50; severe: 51–72). Values of DAS28 < 3.2 indicated low activity disease, whereas values >5.1 were consistent with high disease activity (40, 41). The activity score of DAS28, which assesses exclusively joint involvement, was used to evaluate the severity of PsA (41, 42). Current psoriasis activity was determined by the Psoriasis Area and Severity Index (PASI) which combines severity (erythema, induration, and desquamation) and extension of lesions (% of affected area) (43).

#### Subclinical Marker: cIMT

Ultrasound images of cIMT were recorded within the month of PsA diagnosis. cIMT was defined as the distance (cm) between the inner echogenic line representing the intima–blood interface and the outer echogenic line representing the adventitia-media junction. It was measured at the right and left branches, and the highest value was considered. The distance from the posterior wall of the common carotid arteries was measured at 1 cm from the carotid bulb (bifurcation into external and internal branches). Carotid plaques were also measured (Philips EnVisor C HD Ultrasound Transducer). Values of IMT >0.85 mm indicate atherosclerosis and >1.50 mm are consistent with atheromatous plaques.

#### Non-Conventional and Conventional Cardiovascular Risk Factors

Nonconventional CVRF include proinflammatory cytokines, endothelial activation markers, and CRP. General unspecific inflammatory markers were measured using Commercial enzyme-linked immunosorbent assay (ELISA) kits, according to the manufacturer’s technical specifications. Following 12 h overnight fasting, blood samples were collected and analyzed for glucose, cholesterol, uric acid, lipid profile, and apolipoprotein B. Routine determinations were performed using standard kits (Roche Products).

#### High-Sensitivity C-Reactive Protein (hs-CRP)

The hs-CRP ELISA from GenWay Biotech, Inc. (San Diego, CA, USA) was used to measure total CRP serum levels. Detection limit was 0.0002 mg/mL. Samples with high levels were diluted and re-assayed. In 2013, the American Heart Association and US Centers for Disease Control and Prevention (1913) defined risk groups according to hs-CRP values (mg/L): normal < 0.8, low risk < 1.00, average risk: 1.00–3.00, and high-risk > 3.00. Measuring hs-CRP is only recommended when Framingham clinical score is >5 and <10 years.

#### Interleukin 6 (IL-6)

An *in vitro* ELISA for the quantitative measurement of human IL-6 in serum was performed using the human IL-6 ELISA kit (BD Biosciences, San Jose, CA, USA). The detection limit was 4.7pg/mL.

#### Tumor Necrosis Factor Alpha (TNF-α)

The human TNF-α ELISA kit (BD Biosciences, NJ, USA) was used to quantify TNF-α level. The detection limit was 7.8 pg/mL.

#### Monocyte Chemoattractant Protein-1 (MCP-1)

The concentration of MCP-1 was measured using the Human Quantikine MCP-1 ELISA. The detection limit was 2.31 pg/mL.

#### Soluble Intercellular Adhesion Molecule-1 (sICAM-1)

The Human Quantikine sICAM-1/CD54 ELISA kit was used to determine sICAM-1 levels in serum (R&D Systems, Minneapolis, USA). The detection limit was 1.56 ng/mL.

### Statistical Analysis

Continuous variables were explored for normality and equality of variance (Levene’s test). Results were submitted to the analysis of variance (ANOVA) with Dunnett’s *post hoc* test for between-group comparison and expressed as mean ± SD. Non-parametric data were analyzed using the Kruskal–Wallis test (for variance) and the Dunn’s test for multiple comparisons. A value of *p* < 0.05 was considered statistically significant. Analyses were performed using Instat version 5.0 for Windows (GraphPad Software, La Jolla CA, USA). Plots show individual data instead of the typical mean and SD parameters used for large samples, due to the relatively small number of patients in our study.

## Results

### Demographical Data and Lipid Measurements

Following the selection process, 32 patients entered the study. Three groups were identified: C (9 control healthy patients, 8:1 male-female ratio), CPs (9 low-intermediate CPs patients, 6:3 male-female ratio), and PsA (14 patients with recent PsA onset, 11:3 male-female ratio). The groups were not different according to age, BMI, gender and conventional cardiovascular risk factors (Table 1). Compared with control patients, the CPs group showed increase in TG (48%, *p* < 0.05) and ApoB100 (21%, *p* < 0.05) level, Castelli index (*p* < 0.05), and the ApoB100/Apo A1 ratio (*p* < 0.05) (Table 1). The PsA group showed higher Castelli index (*p* < 0.05) and ApoB100/Apo A1 ratio (*p* < 0.05) compared with control patients (Table 1).

**Table 1:** Demographical data and blood lipid profile.

Factor	Control (*n* = 9)	CPs (*n* = 9)	PsA (*n* = 14)
Age	45.3 ± 16.4	50.3 ± 7.5	49.4 ± 18.5
Male, *n* (%)	8 (88)	6 (66)	11(78)
Caucasian, *n* (%)	9 (100)	9 (100)	14 (100)
BMI (kg/m^2^)	25.9 ± 5.7	27.0 ± 2.0	29.1 ± 4.6
Total cholesterol (mmol/L)	183.6 ± 38.9	211.2 ± 41.7	174.9 ± 35.9
Triglyceride (mmol/L) (1)	83.25 ± 8.3	123.4 ± 0.3*	99.5 ± 53.0
HDL (mmol/L)	51.0 ± 21.4	43.2 ± 16.0	33.55 ± 9.1
LDL (mmol/L)	103.5 ± 34.7	137.1 ± 27.0	108.1 ± 35.3
Apo B100 (mg/dL)	103.8 ± 8.1	125.6 ± 7.1*	104.8 ± 9
Apo A1 (mg/dL)	151.6 ± 7.2	155.4 ± 7.2	143.7 ± 9
Castelli Index	3.93 ± 0.54	4.80 ± 0.53*	4.80 ± 0.31*
Apo B100/Apo A1	0.58 ± 0.16	0.85 ± 0.30*	0.70 ± 0.15*

*Symptoms began by 1–2 months’ average after the time of the investigation. Control: healthy subjects*.

*p < 0.05 vs. control.

*CPs, cutaneous psoriasis group; PsA, psoriatic arthritis group; n, number of patients; BMI, body mass index*.

### Inflammatory Biomarkers

**Figure 1:**
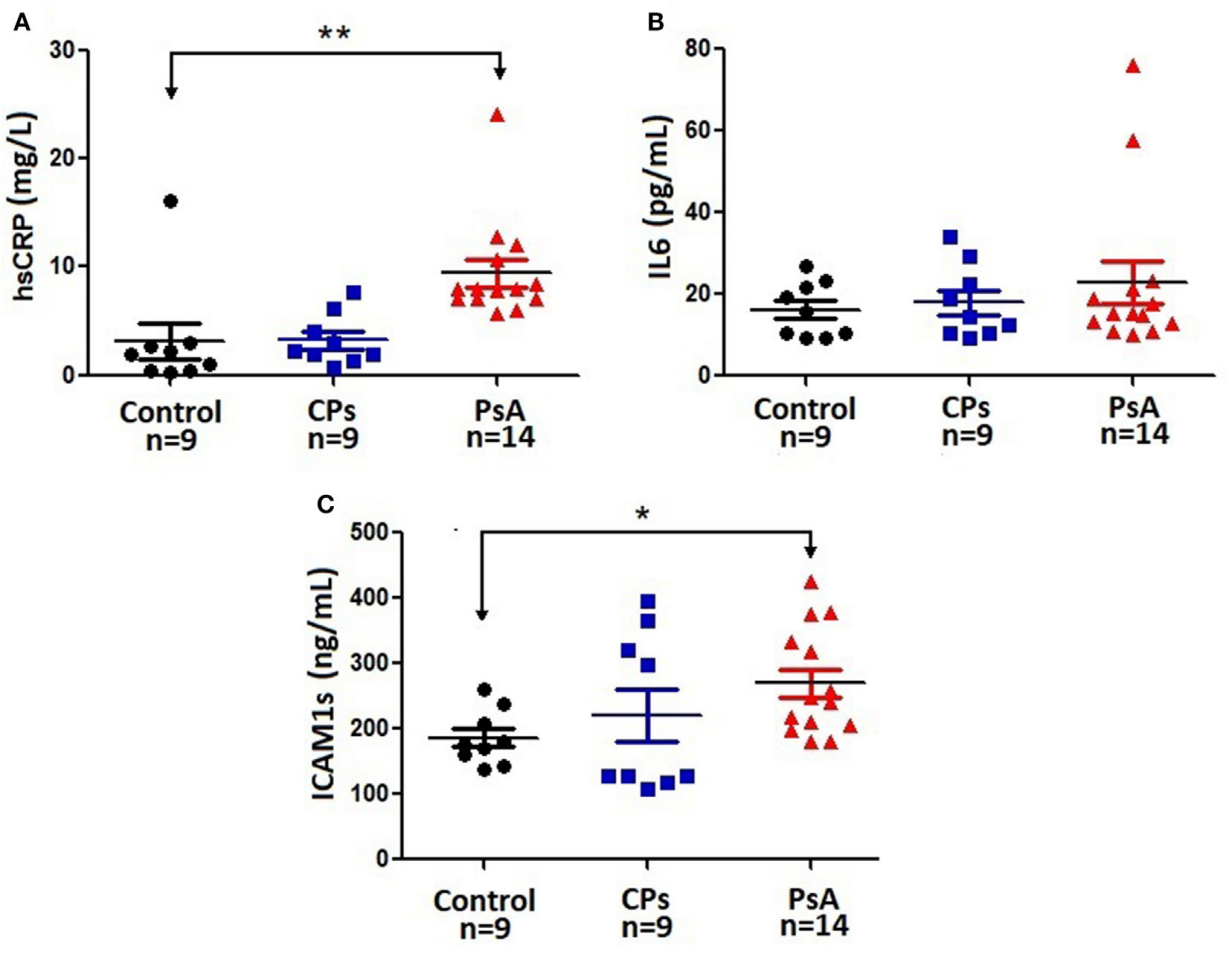
Serum level of iinflammatory biomarkers. Individual data are plotted along with the typical mean and SD parameters due to the relatively small number of patients in this study. Control: healthy subjects. CPs, cutaneous psoriasis group; PsA, psoriatic arthritis group; *n*, number of patients. *p < 0.05, **p < 0.01 between indicated groups. **(A)**: hs-CRP, high-sensitivity C-reactive protein; **(B)**: IL-6, interleukin-6; **(C)**: sICAM-1, soluble intercellular adhesion molecule-1.

The PsA group showed a dramatic increase in hs-CRP level compared with both C (*p* < 0.01) and CPs (*p* < 0.05) groups (Figure 1A). Also, the level of sICAM1 increased by 54% (*p* < 0.05) in PsA compared with control patients. Other inflammatory biomarkers as IL-6 (Figure 1), TNF-α (2.2 ± 0.9 in C, 1.4 ± 0.7 in CPs, 3.6 ± 2.1 in PsA) or MCP-1 (142.2 ± 7.6 in C, 160.5 ± 14.4 in CPs, 142.3 ± 11.5 in PsA) did not change in any group. As expected, the erythrocyte sedimentation rate (ESR, mm/h) increased in both CPs (85.8 ± 9.1, *p* < 0.01) and PsA (53.0 ± 17.7, *p* < 0.01) groups compared with control patients (12.7 ± 5.1).

### Carotid Intima–Media Thickness

**Figure 2:**
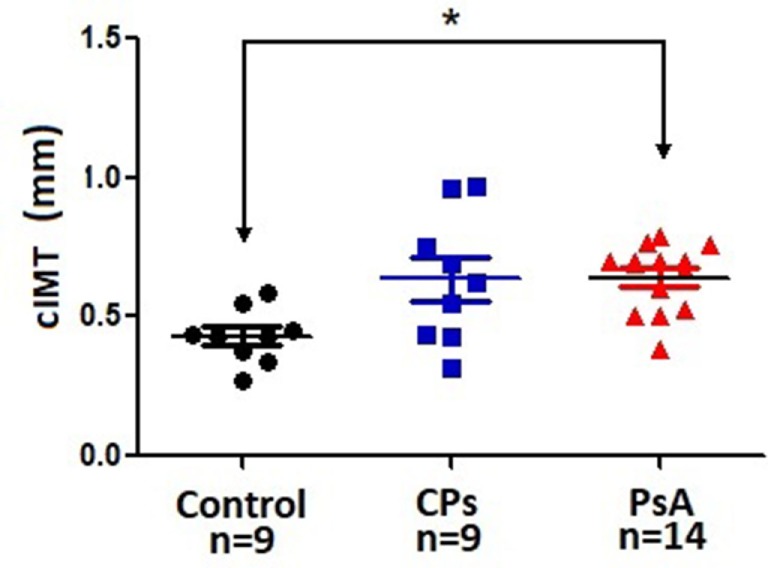
Carotid IMT. Individual data are plotted along with the typical mean and SD parameters due to the relatively small number of patients in this study. Control: healthy subjects. CPs, cutaneous psoriasis group; PsA, psoriatic arthritis group; *n*, number of patients. *p < 0.05 between indicated groups. IMT, intima–media thickness.

**Figure 3:**
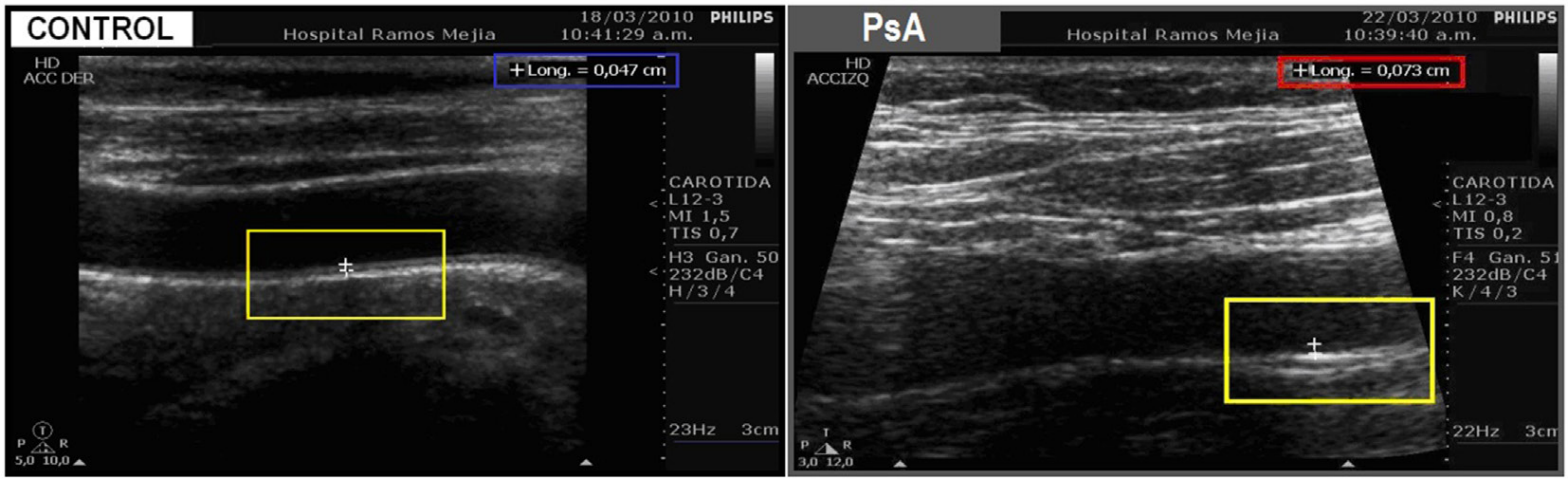
Representative echocardiography images of control patients (left) and PsA patients (right). The left image shows a normal carotid wall with normal intima/media thickness (cIMT = 0.47 mm) in a control patient. Compared with the control image, the right image clearly shows carotid wall thickening (cIMT = 0.73 mm) in a PsA patient.

Only PsA patients (0.590 ± 0.045), unlike CPs patients (0.621 ± 0.100), showed higher cIMT values compared with controls (0.436 ± 0.051) (Table 2; Figures 2 and 3).

**Table 2:** Inflammatory markers and characteristics of carotid artery wall.

Variable (mean ± SD)	Control (*n* = 9)	CPs (*n* = 9)	PsA (*n* = 14)
ESR (mm/h)	12.7 ± 5.1	85.8 ± 9.1**	53.0 ± 17.7**
sICAM-1 (ng/mL)	174.8 ± 11.9	221.2 ± 40.2	269.3 ± 2.4*
IL-6 (pg/mL)	16.3 ± 2.5	18 ± 2.9	23.3 ± 5.5
hs-CRP (mg/L)	3.15 ± 1.85	3.26 ± 0.76	8.4 ± 4.4**
TNF-α (pg/mL)	2.1 ± 1.0	1.4 ± 0.7	3.6 ± 2.1
MCP-1 (pg/mL)	142.2 ± 7.6	160.5 ± 14.4	142.3 ± 11.5
Mean cIMT (mm)	0.436 ± 0.051	0.621 ± 0.100	0.590 ± 0.045*
Plaques (No./total) and (%)	0 (0); 0	3 (3/9); 33	1 (1/14); 7.14

*Control: healthy subjects. Data are expressed as mean ± SD*.

*p < 0.05 vs. control.

**p < 0.01 vs. control.

*CPs, cutaneous psoriasis group; PsA, psoriatic arthritis group; *n*, number of patients; ESR, erythrocyte sedimentation rate (normal: ≤22 mm/h in men, ≤29 mm/h in women); cIMT, carotid intima–media thickness;IL-6, interleukin-6; sICAM-1, soluble intercellular adhesion molecule-1; hs-CRP, high-sensitivity C-reactive protein; cIMT, intima–media thickness*.

### Psoriasis Area and Severity Index (PASI)

CPs (5.857 ± 0.950) patients showed higher PASI values compared with PsA (3.042 ± 0.951).

## Discussion

In the past decade, the critical role of inflammation in the development of cardiovascular diseases has been accepted. Current evidence suggests that the inflammatory pathway in atherosclerosis culminates in altered concentrations of various markers in peripheral blood (31, 32) including CRP (44), IL-6, TNF-α, MCP-1, and ICAM1 (45–47). In 2013, the European Task Force for cardiovascular prevention and The American Heart Association and US Centers for Disease Control and Prevention (2013) recommended the use of only CRP among inflammatory biomarkers. Recently the Task Force established 24 inflammatory mediators including TNF, IL-6, sICAM-1, and MCP-1, presently accepted as potential surrogate biomarkers of CVR. Despite the supporting literature they have only a marginal use in clinical practice (17, 18).

### Inflammatory and Morphological Subclinical Biomarkers in the Context of Systemic Inflammatory Burden

#### CVR-Related Serologic Biomarkers

Our findings show an increase of nonconventional inflammatory biomarkers of CVR in blood near the onset of symptoms and signs of PsA, even in the absence of conventional CVRFs. Psoriatic patients with no cardiovascular history or CVRFs showed an increase in CRP, sICAM-1 blood levels and cIMT compared with age- and BMI-matched control subjects. Of note, only selected PsA patients progressing from previous low or mild CPs entered the study to assure homogeneity of the inflammatory context without medication.

These data agree with current epidemiologic evidence predicting associated CVR (44), and the early role of these biomarkers in atherosclerosis. Our findings agree with the high level of sICAM-1 and of other inflammatory biomarkers found in matched age and gender psoriatic patients (40). These findings may be interpreted as predictors of future arterial disease (23).

In population studies, serum levels of MCP-1 correlate with CVR factors and with coronary and peripheral artery disease incidence (24). The level of MCP-1 in the blood may be involved in the pathogenesis and activity of Psoriasis vulgaris (48). In our study, we did not observe changes in MCP-1 level in blood.

Results do not allow us to know if systemic concentrations of sICAM1 and MCP-1 derived exclusively from the endothelium or whether TNF-α and IL-6 derived only from immune cells. Different sources as the white adipose tissue, the liver and autoimmune targets like skin and joints contribute to the systemic levels of MCP-1, sICAM1, IL-6, and TNF-α (16).

In turn, a sustained increase in the level of IL-6 and TNF-α favors the hepatic release of acute-phase reactants including CRPs (42), critically involved in PsA, CPs, and atherosclerosis. However, the increase in CRP might be related to increased level of cytokines other than IL-6 which did not show changes in psoriatic patients in our study. We have found an increase in IL-1β level in PsA patients compared with controls (21.60 ± 2.39 vs. 14.64 ± 2.98, respectively, *p* < 0.05) that might account for CRP release. Additionally, in psoriatic patients, the skin releases IL-22 which might also help to explain the increased in CRP level in our study. The release of MCP-1, sICAM1, IL-6, and TNF-α is critically determinant in defining the load of systemic inflammation (16). Our results indicate an enhanced inflammatory burden reflected by carotid wall thickening and higher blood level of sICAM-1 and CRP in PsA patients without conventional risk factors. Pathological metabolic inflammatory activated pathways seem to be necessary to fully release TNF-α and MCP-1 from adipose tissue, limiting blood levels of TNF-α and MCP-1. In our study, TNF-α and MCP-1 differences between psoriatic patients and controls did not reach statistical significance. Recent findings suggest that the nutrient-induced gut hormone gastric inhibitory polypeptide induces adipose tissue inflammation by triggering a crosstalk between adipocytes and macrophages involving MCP-1 (47, 49, 50, 51). Elevation of ICAM-1 released by adipose tissue does not require TNF-α to upregulate its expression. How excess body fat triggers inflammatory-like responses is not clear, but it is thought to involve elevated levels of cytokines (52), ICAM-1 (53, 54), and MCP-1 (50, 52).

#### Intima–Media Thickness

The increase of cIMT is a robust and early indicator of overall atherosclerosis (15). It has been used as a surrogate marker of subclinical atherosclerosis and considered a non-invasive diagnostic tool for identification of premature atherosclerosis. Our study indicates an increase in cIMT suggesting a proatherogenic condition in psoriatic patients with higher systemic levels of inflammatory biomarkers and absence of conventional CVRFs.

Due to the exclusion criteria in this study, total systemic inflammation likely resulted from limited adipose tissue inflammation or metabolic load and from low to mild inflammation of the skin and joints according to PASI and DAS28 score or autoimmune load. In previous studies, patients developed hypertriglyceridemia and showed an increase in the ApoB100/ApoA1 ratio, both atherogenic and increasing cardiovascular risk (55). Others have also reported changes in HDL levels (56). Present findings agree with the positive association reported for the ApoB/ApoA-I ratio with cIMT, suggested as a suitable tool for early detection of premature atherosclerosis in psoriasis patients (17, 18). One study reported a higher prevalence of triglycerides and lower prevalence of low HDL levels in Ps patients (56).

Integrative interpretation of a subset of measures, e.g., cIMT, CRP, and sICAM-1, and calculation of the ApoB100/ApoA1 ratio might help in the early estimation of premature atherosclerosis in psoriatic patients. Carotid wall thickening is related to conventional CVRFs like age, BMI, and the level of TG, LDL-cholesterol, and total cholesterol in blood (11, 57).

The presence of carotid plaques correlated with hypertriglyceridemia and psoriasis activity (PASI and DAS28 values) (58). Qu et al. reported that cIMT increases with the load of classical risk factors and with the level of CRP (59).

Notwithstanding the exclusion of hypertriglyceridemia in the selection process prestudy, CPs patients showed higher TG level compared with either control of PsA patients.

To date, CRP appears to be the only inflammatory marker used as a potential predictor of overall atherosclerosis. Even so, measuring CRP level is still discussed in the general population and even in the context of IMID.

In this study, the level of some biomarkers of proatherogenesis did not follow the patients’ clinical status what may seem at first counterintuitive. Although the level of sICAM-1, IL-6, hs-CRP, and TNF-α appeared to be higher in PsA than in CPs patients, ESR, MCP-1, and probably also cIMT apparently developed in the opposite direction as CPs patients showed higher values compared with PsA patients. One possible explanation is that ESR, MCP-1, and cIMT might be more sensitive to the activity of the disease as evidenced by PASI values. Then, a large skin damage (a higher PASI index) might be crucial in raising the value of the ESR, and the level of MCP-1 and cIMT in CPs patients. Also, the level of ESR, MCP-1, and cIMT might be more sensitive to traditional CV risk lipid factors. Within normal values, the CPs group showed higher level of total cholesterol, TG, and LDL-cholesterol than the PsA group.

### Atherosclerosis Tendency

In sum, patients with recent-onset PsA showed an increase in cIMT and blood level of sICAM-1 and CRP compared with healthy controls in our study. In low-mild CPs patients, CRP and sICAM-1 levels were not different from those found in healthy controls. The level of IL-6 showed a mild increasing trend with the severity and clinical expression of the disease, though between-group differences were not statistically significant. Likewise, the groups were indistinguishable based on the level of MCP-1 and TNF-α.

Psoriasis target tissues like skin and joints critically contribute to systemic inflammation. The strict exclusion criteria limited the conventional cardiovascular risk factors which nevertheless likely contributed to the overall inflammatory burden.

It is time to obtain a new built-in CVR index considering both compartments. The IMIDs may need special consideration due to the intrinsic and chronic autoimmune inflammation. Certainly, both conventional and non-conventional CVRFs contribute to systemic inflammation and interact with each other inducing the final systemic load of inflammation (16).

## Conclusion

Our results suggest that recently diagnosed non-medicated PsA patients without conventional CV risk factors develop subtle chronic inflammatory changes as the observed increase in sICAM1, and CRP which might foster microenvironmental conditions for carotid wall thickening.

Recent onset PsA patients no medicated develop subclinical atherosclerosis compared with controls compared with patients with low-mild (no medicated) CPs. The degree of atherosclerosis tendency may be related to the amount of the inflammatory burden suggesting an ongoing subclinical inflammation either previous or concurrent with joints’ damage. The release of autoimmune inflammatory mediators may account for the increase in systemic inflammatory burden in PsA and CPs patients with low disease activity in our study. However, the amount of metabolic load contributes to fully expressing proatherogenic inflammation.

Psoriatic patients are known to have a high level of TG and low level of HDL even within normal values compared with healthy subjects as observed in our study. The ApoB100/ApoAI ratio and Castelli index reflect the counterbalance between proatherogenic and antiatherogenic lipoproteins (39) related to CVR. The former seems to be a better predictor of cardiovascular disease than the latter (60).

The strict exclusion criteria for conventional CVRFs resulted in a long time of recruitment and a relatively small number of subjects. We encourage further research including a larger number of patients to confirm present information.

### Study Limitations

Sample size is very small. Recruiting patients complying with the inclusion and exclusion criteria required for our study took us several years. One major handicap limiting the sample size was the essential requirement on the absence of traditional cardiovascular risk factors in patients around their fifties. Also, the seemingly counterintuitive mismatch between the level of some biomarkers of proatherogenesis and the patients’ clinical status deserves further review.

## Ethics Statement

This study was carried out in accordance with the recommendations of the respective Institutional Ethics Committees of the Faculty of Medicine of the University of Buenos Aires (Comité de Bioética de la Facultad de Medicina de la Universidad de Buenos Aires) and of the Hospital JM Ramos Mejía (Comité de ética en investigación del Hospital José María Ramos Mejía del Gobierno de la Ciudad de Buenos Aires, Argentina, registered at The Office for Human Research Protections, NIH, USA Code FWA 00001767). All subjects gave written informed consent in accordance with the Declaration of Helsinki. The protocol was approved by the committees mentioned above.

## Author Contributions

Design: RF and FC. Data collection: RF, VC, JT, EK, AU, MC, and NK. Analysis: RF, MO-L. Manuscript writing: RF, FC, and MO-L.

## Conflict of Interest Statement

The authors declare that the research was conducted in the absence of any commercial or financial relationships that could be construed as a potential conflict of interest.
